# A Comparative Study of Contemporary Color Tongue Image Extraction Methods Based on HSI

**DOI:** 10.1155/2014/534507

**Published:** 2014-11-20

**Authors:** Mingfeng Zhu, Jianqiang Du, Chenghua Ding

**Affiliations:** ^1^School of Computer Science, Jiangxi University of Traditional Chinese Medicine, Nanchang 330004, China; ^2^School of Fundamental Medical Science, Jiangxi University of Traditional Chinese Medicine, Nanchang 330004, China

## Abstract

Tongue image with coating is of important clinical diagnostic meaning, but traditional tongue image extraction method is not competent for extraction of tongue image with thick coating. In this paper, a novel method is suggested, which applies multiobjective greedy rules and makes fusion of color and space information in order to extract tongue image accurately. A comparative study of several contemporary tongue image extraction methods is also made from the aspects of accuracy and efficiency. As the experimental results show, geodesic active contour is quite slow and not accurate, the other 3 methods achieve fairly good segmentation results except in the case of the tongue with thick coating, our method achieves ideal segmentation results whatever types of tongue images are, and efficiency of our method is acceptable for the application of quantitative check of tongue image.

## 1. Introduction

Tongue diagnosis is one of the important contents of “Four Diagnoses” in Traditional Chinese Medicine (TCM). The “Four Diagnoses” means observation, listening, interrogation, and pulse-taking. Traditional tongue diagnoses depend on observations of tongue features such as color, shape, moisture, and texture by TCM doctors. The results of tongue diagnoses are influenced not only by the experience of TCM doctors but also by the surrounding environments. Therefore, nowadays many researchers use digital camera to take tongue photos and utilize computer to make quantitative checks and analyses of tongue images, that is, objectification of tongue diagnoses. To check and analyze tongue images quantitatively, we first need to segment tongue body region out of the background which is so called tongue image extraction. And automatic and accurate extraction of tongue image is an important sign of intelligent analysis of tongue images.

In recent years, various kinds of image segmentation methods have been applied to the application of tongue image extraction. Among these methods, the representative ones are active contour method, level sets method, region growing and merging, random walk method, and so forth.

Active contour method which was so called Snakes model was widely used in tongue image extraction application. Shi et al. [1] utilized geodesic active contour model to make tongue image segmentation. The proposed approach could enhance the accuracy and practicability obviously, compared with other work. When the surface of the tongue image was not regular, this might lead to a failure to extract tongue body region out of the background successfully. Shi et al. [2] presented a fully automated active contour initial method that utilized prior knowledge of the tongue shape and its location in tongue images. This method increased the curve velocity but decreased the complexity. The only inconvenience of this method is that 4 points were needed to specify in order to build initial contour. Liang and Shi [3] proposed a new tongue segmentation approach based on the combination of the feature of tongue shape and the Snakes correction model. In this method a rough tongue contour was got using the features of tongue image in HSI color model. The experimental results showed this method was efficient in the case of tongue images given by the authors, but the number of the experimental samples was quite limited. Zhai et al. [4] transformed tongue image into HSI color model and dual Snake algorithm was used to obtain the accurate contour of the tongue body. Through testing, this method had proved to be satisfactory for the specific tongue image segmentation. But the initial inside contour and initial outside contour when implementing the dual Snake algorithm were difficult to obtain, so this method is quite theoretical from a certain point of view. Ning et al. [5] presented an automatic tongue segmentation method which used a region merging method to make segmentation and utilized Snakes algorithm to refine the region merging result. The proposed method greatly enhanced the segmentation performance, but the accuracy of this method was not high when processing some tongue image samples given by the authors. Li [6] suggested a kind of tongue image extraction method using improved Snakes model. Through the minimum calculation of Snakes model, estimated contour line was further processed which could improve the accuracy of tongue image extraction, but the efficiency of the proposed method is not mentioned in the article. Wang et al. [7] proposed an improved tongue image extraction approach based on Snakes model, in which the tongue image was described in two other color space and a two-step Snakes implementation was used. The accuracy and reliability of this method were improved, but the efficiency of this method might be quite low due to the processing with high complexity using this method. Fu et al. [8] used radial edge detection to get rough contour of the tongue image, utilized pair-color-remove to remove the lip, and applied Snakes method to get the exact contour of the tongue. The accuracy of this method was proved in the experiment, but the efficiency of this method was not mentioned in the article.

Level sets and random walk methods also brought enough attention in the application of tongue image extraction by related researchers. Zhu and Du [9] introduced a kind of color tongue image fast segmentation method based on level sets, in which the boundary feature weight function was improved and a kind of variable time step method was introduced. Both accuracy and efficiency were improved greatly, compared with the traditional level sets method. But when the surface of tongue body region was not regular, the segmentation effect of this method might not be very ideal. Li et al. [10] proposed a novel method for tongue contour extraction based on improved level set curve evolution, in which an automatic initialization of contour was presented and both the color information and tongue contour shape were used to segment tongue images. Applying this method to the large database of tongue images, promising experimental results were achieved. But the efficiency of this method was not mentioned in the article. Zhu and Du [11] suggested a kind of improved random walk algorithm and applied it to color tongue image segmentation, in which toboggan algorithm was adopted to segment original tongue image into initial regions; a newly designed weighted-graph was built and random walk algorithm was applied to make final segmentation. Both the accuracy and efficiency of this method were greatly improved, compared with traditional random walk method. But for the tongue image with irregular coating on its surface it might lead to a failure to segment tongue body region successfully.

Other kinds of methods enriched the application of tongue image extraction. Zhu et al. [12] suggested a kind of color tongue image extraction method which utilized greedy rules with fusion of color and space information in order to extract tongue body region from background accurately. The accuracy of this method was quite high. In particular, for those tongue images with coating, this method could achieve relatively good segmentation effects. And the efficiency of this method was acceptable and practical for the applications of tongue diagnoses. Xu et al. [13] proposed a fully automatic tongue detection and tongue segmentation framework. Compared with other existing methods, this framework was fully automatic without any need of adjusting parameters for different images and did not need any initialization. But there was only one sample in the experiment, so it was not enough to prove the accuracy of this method for various kinds of tongue images. Zhong et al. [14] suggested a novel method to segment the tongue image automatically with the mouth location method and active appearance model. Due to the different positions of tongues in the tongue images, this method needed to use different initial contours to segment tongue body region, which brought some inconvenience to the applications of tongue diagnoses. Yang et al. [15] proposed an image segmentation algorithm based on the shortest path. The theoretical basis was quite detailed, but the accuracy of this method was not completely proved according to the experimental results and the efficiency of this method was not mentioned in the article. Li et al. [16] studied a new theory of fuzzy rough sets and presented a method for segmenting tongue images, which extracted condensation points by the theory of fuzzy rough sets, quartered the data space layer by layer, and softened the edge of the dense block by drawing condensation points in the borders. The application result indicated that the algorithm could avoid segmenting image excessively and speed up segmentation velocity by fuzzy grid dividing. Nevertheless, the number of the sample in the experiment is only one, so that it is not enough to prove the wide practicability of this method in the applications of tongue diagnoses. Zhong et al. [17] suggested a kind of new method for segmenting the tooth-marked tongue images, which converted RGB color space into HSI color space and used Otsu threshold value to complete segmentation of tooth-marked tongue images. It was mentioned in the article that the speed of the method was quick and the accuracy of the method was high, but no enough evidences were provided in the article. Chen et al. [18] combined one of the graph theory image segmentation methods and multiresolution image segmentation together to segment the image on two different resolutions. The proposed method was novel, but the accuracy was not high which was only 87.3% and the efficiency test was not provided by the authors. Zhang and Qin [19] designed a new method for tongue image segmentation, which combined gray projection and threshold-adaptive method to segment tongue images. The experimental results of this method were fairly good, but the comparisons with other methods in accuracy and efficiency were lacking. Li and Wei [20] proposed an adaptive segmentation algorithm to segment tongue images efficiently, which divided tongue image into several parts, used an iterative approach to calculate each subblock threshold, and used each local threshold to segment tongue images. The experimental results showed that the algorithm could segment well the tongue images whose background and boundaries were not clear. But only 2 samples which were a tongue image with withered coating and a tongue image with white coating were processed and the effectiveness of this method was not proved by the limited number of samples. Zhao et al. [21] utilized mathematical morphology to describe shape features of images, which was combined with HSI color model to segment tongue images. The effect of this method for the tongue without coating was fairly good, but if there was thick coating on the surface of tongue body region, it might lead to a failure to segment tongue body region successfully. Du et al. [22] suggested a kind of color tongue image segmentation algorithm based on HSI model in which original images were converted into HSI color space, tongue images were segmented by threshold values of hue, and intensity and sequential algorithm was used to mark the connected regions. The experimental results of this method were quite good for those tongue images the surfaces of which were regular, but when the surfaces of tongue body regions were not regular, it might fail to segment tongue body region successfully.

In the methods [3, 4, 9, 11, 12, 17, 21, 22] mentioned above, there were a common ground that they were using HSI color model to describe the features of tongue image. And HSI color model is closer to human vision and due to specificity of the application of tongue image extraction using HSI color model can achieve better segmentation results than other methods. In addition, tongue coating is of important meaning in TCM clinical tongue diagnoses and to extract tongue image with coating is of a certain difficulty. Therefore, we designed and implemented a kind of tongue image extraction method which utilized multiobjective greedy rules and made fusion of color and space information to extract tongue body region in HSI color model. Owing to the fusion of color and space information, this method could extract tongue image with coating accurately, which other methods could not be competent to achieve. From now on, we will discuss HSI color model and the principle of tongue image extraction in HSI color model as well as compare the typical method [21], method [22], and Snakes method with our recent tongue image extraction method suggested in a new China invention patent.

## 2. Materials and Methods

### 2.1. HSI Color Model

**Figure 1:**
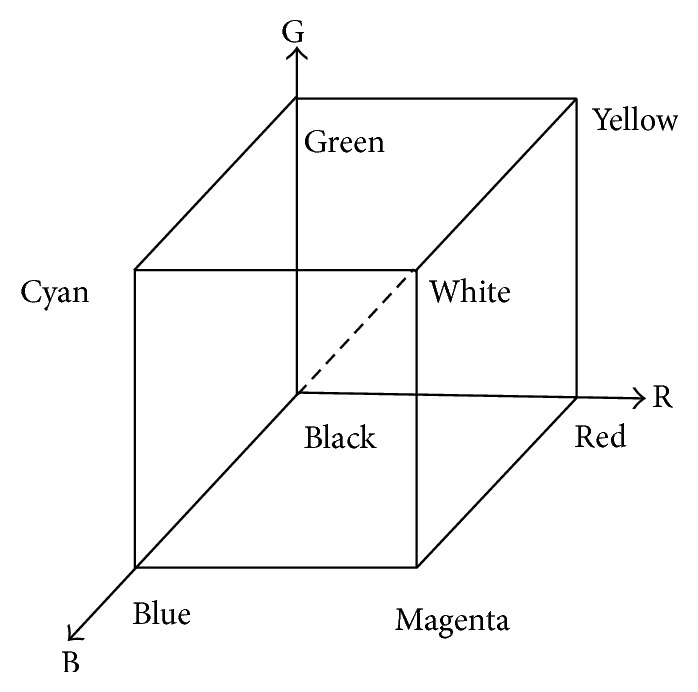
RGB color model.

A static image is commonly expressed in a 2-dimensional pixel matrix. Each pixel is composed of 3 colors, that is, red, green, and blue. So RGB color model is feasible and suitable to express and store static images. RGB color model can be denoted in Figure 1.

**Figure 2:**
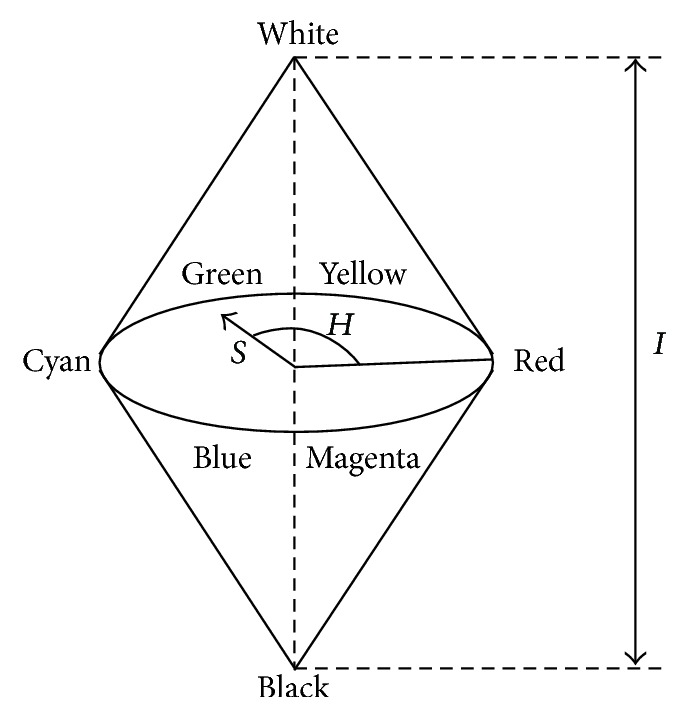
HSI color model.

HSI color model uses hue, saturation, and intensity 3 elements to describe the features of images, which is closer to perception principle of human vision. Herein, hue is the color type of a pixel, saturation is the degree to which a certain color is mixed into other colors, and intensity is the brightness of a pixel. HSI color model can be denoted as Figure 2. The formulae of hue, saturation, and intensity are shown in formula (1), formula (2), and formula (3) respectively. Consider

(1) $$\begin{array}{c} H=\text{arccos}\left\{\frac{\left[\left(R-G\right)+\left(R-B\right)\right]/2}{{\left[{\left(R-G\right)}^{2}+\left(R-B\right)\left(G-B\right)\right]}^{1/2}}\right\}, \end{array}$$

(2) $$\begin{array}{c} S=1-\frac{3}{\left(R+G+B\right)}\left[\min\left(R,G,B\right)\right], \end{array}$$

(3) $$\begin{array}{c} I=\frac{1}{3}\left(R+G+B\right). \end{array}$$

### 2.2. Principle of Tongue Image Extraction Based on HSI Color Model

**Figure 3:**
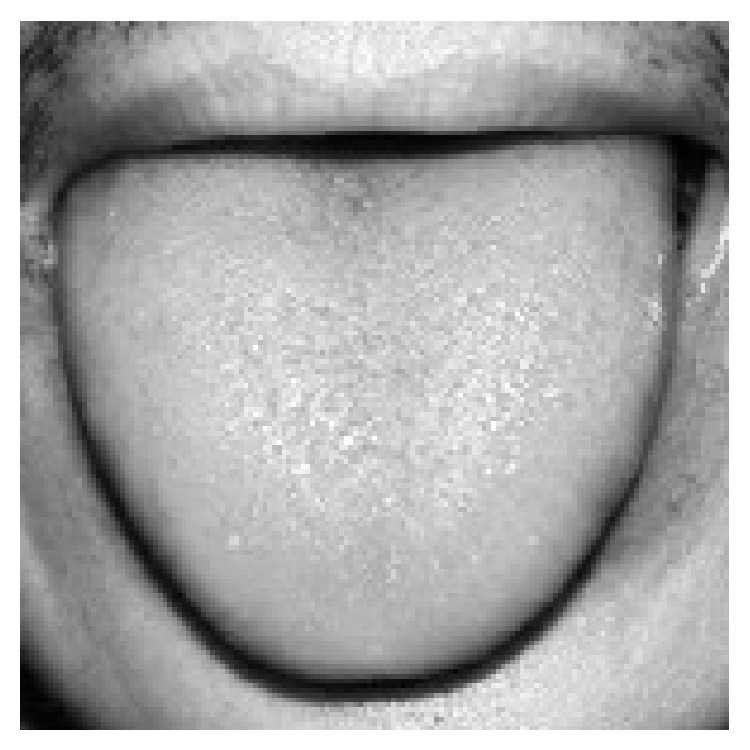
Grayscale image of tongue.

**Figure 4:**
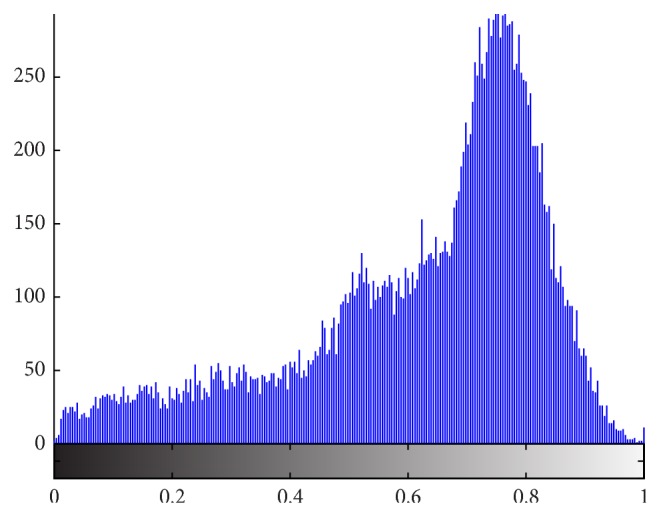
Grayscale histogram of tongue.

In many traditional segmentation methods, intensity is the only feature to decide whether it is the object or background in the image. Even if it feasible in many segmentation applications, as far as tongue image extraction is concerned, using intensity information is not enough to segment tongue body region out of background. As we can see from Figure 3, the intensity of tongue body region and that of face region are identical. And in Figure 4, which is the grayscale histogram of tongue, there is only one peak, which represents the face and tongue body regions. Therefore, we can not distinguish tongue body region from face region only by intensity information.

The main hue of tongue body region is red and entire tongue body region is connected. The hue of surrounding region (such as face, teeth) is different from that of tongue body region, except the mouth lip region which is connected to tongue body region. Nevertheless, the intensity of the boundary between tongue body region and mouth lip region is relatively low compared to that of tongue body region and mouth lip region. Therefore, it is possible to separate tongue body region out of the surrounding regions by hue and intensity information.

**Figure 5:**
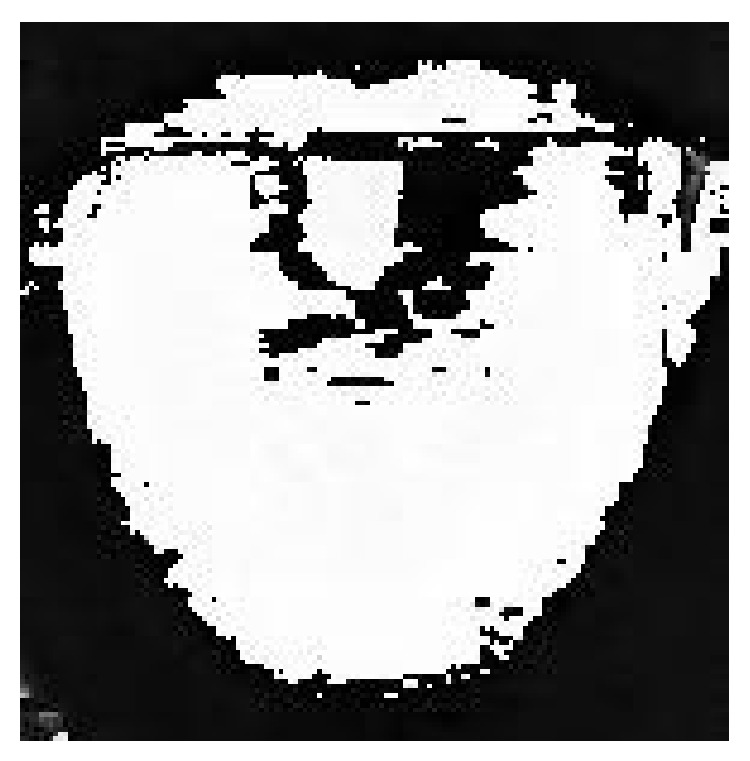
Hue image of tongue.

**Figure 6:**
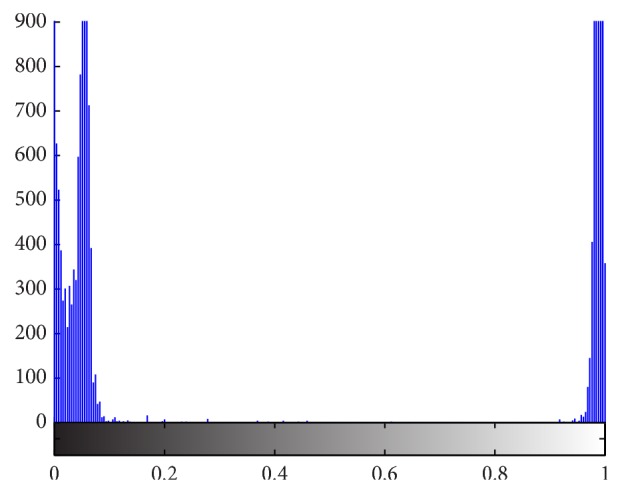
Hue histogram of tongue.

Traditional threshold segmentation is based on analyses of histogram and the main feature of tongue body region is its red hue. In hue image of tongue, there are not only pixels with high values but also pixels with low values on the tongue body region as Figure 5 shows. In hue histogram of tongue image, the red hue lies on the start and the end of it, as we can see in Figure 6. The hue distribution of the histogram conforms to the hue image of tongue.

**Figure 7:**
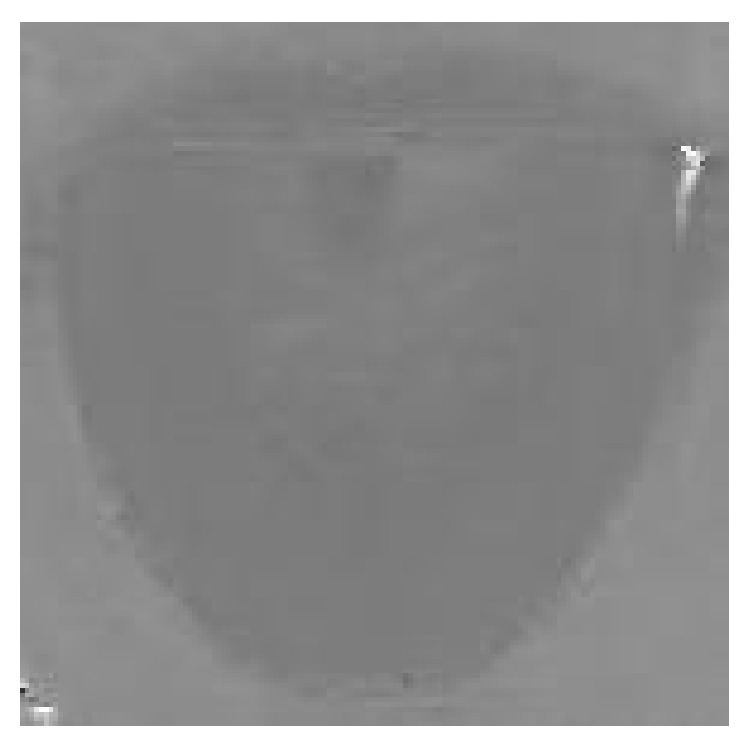
Hue image after transformation.

**Figure 8:**
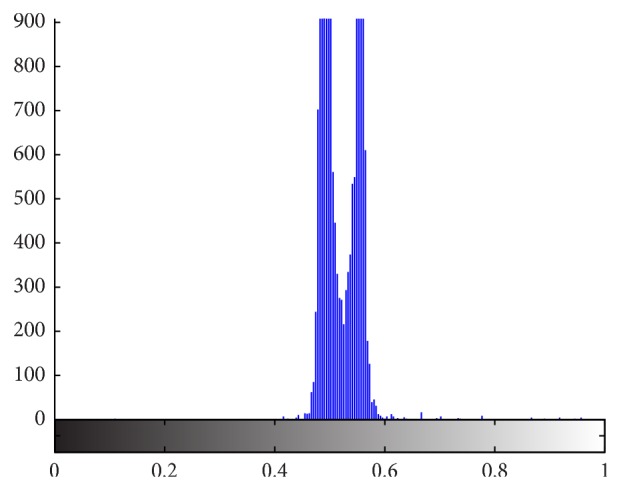
Hue histogram after transformation.

In hue histogram of tongue, it seems that there are 3 peaks in it. The range of hue histogram is from 0 degree to 360 degree; that is, 360 degree is its cycle. In fact, the start and the end of hue histogram are adjacent. In order to make 0 degree and 360 degree adjacent in hue histogram, we need to make some transformations to the histogram. The concrete method is to move the part from 180 degree to 360 degree to the left of the histogram and move the part from 0 to 179 to the right of the histogram. In this way, 0 degree and 360 degree in the histogram are adjacent in the hue histogram. The hue image of tongue after transformation is shown in Figure 7 and the corresponding hue histogram is shown in Figure 8.

From Figure 8, we can see 2 obvious peaks in the hue histogram. According to Ostu's theory, it is feasible to segment the object region from the background by the valley threshold. And as we mention above, even if the tongue body region and mouth lip are connected, the intensity of the boundary between them are relatively low. Therefore, using hue and intensity information we can separate tongue body region out of background successfully. Now, we introduce 2 typical kinds of tongue image extraction method based on HSI color model.

### 2.3. Method Based on Mathematical Morphology and HSI Color Model

Zhao et al. [21] suggested a kind of color tongue image segmentation method based on mathematical morphology and HSI. The principle and procedure are given as follows.

**Step 1 .**

Convert original tongue image from RGB color space into HSI color space.

**Step 2 .**

Use hue information to make binarization of the tongue image.

**Step 3 .**

Use clustering algorithm to make object region clustering.

**Step 4 .**

Use mathematical morphology such as opening and closing to remove small holes on tongue body region.

This method is the most typical and earliest color tongue image extraction method based on HSI. This method is efficient, but it can only handle the tongue image without coating. Later, we will discuss the experimental effect of this method.

### 2.4. Method Based on Sequential Algorithm and HSI Color Model

Du et al. [22] introduced a kind of color tongue image segmentation method based on sequential algorithm and HSI color model. This method is an improved method of the method mentioned in [21]. Its principle and procedure are given as follows.

**Step 1 .**

Convert original tongue image F0 from RGB color space into HSI color space.

**Step 2 .**

Segment tongue image using hue and intensity information and image F1 with mouth and tongue body regions is obtained.

**Step 3 .**

Sequential algorithm is used to segment tongue image F1 and the tongue body region in image A1 is obtained.

**Step 4 .**

Mathematical morphology closing operation is applied for A1 to fill small holes on the tongue body region and image A2 is obtained.

**Step 5 .**

Image A2 is multiplied by original image F0 to gain the target tongue image successfully.

Compared with the method [21], this method can get more accurate segmentation results. But when it comes to a tongue image with very large size, this method may be a little bit slow. The same as the method mentioned in [21], this method cannot handle the tongue image with thick tongue coating well. Later, the practical effect of this method will be illustrated in detail.

### 2.5. Method with Fusion of Color and Space Information by Applying Multiobjective Greedy Rules

Zhu et al. [12] introduced a novel approach for color tongue image extraction with fusion of color and space information, which was suggested in a recent authorized China invention patent. This method used multiobjective greedy rules and made fusion of color and space information to extract tongue image. HSI color model was used to describe the color features, in which both hue and intensity were utilized. Due to introduction of space information, this method showed a great advantage over other methods in which this method could extract tongue image with coating accurately. As mentioned before, tongue image with coating is of important clinical diagnostic meaning; for example, white tongue coating indicates exterior syndrome and cold syndrome, and yellow tongue coating indicates heat syndrome and interior syndrome.

There are 4 multiobjective greedy rules in this algorithm, which are denoted as follows.

**Rule 1 .**

The pixels in the target region are tongue substance.

**Rule 2 .**

If the pixels in the target region are not tongue substance, they must be tongue coating, which are surrounded by the pixels of tongue substance.

**Rule 3 .**

Each included pixel in the target region must be tongue substance or tongue coating; otherwise, it is abandoned.

**Rule 4 .**

The target region is the largest connected region.

From the 4 rules mentioned above, we can know that the goal of this algorithm is to find the largest region most similar to tongue substance and tongue coating features.

By applying the 4 rules mentioned above, the procedure of this algorithm can be denoted as follows.

**Step 1 .**

Set initial target region *S* to null.

**Step 2 .**

If the intensity of start pixel *x* is high and its hue is close to tongue substance, pixel *x* is included in target region *S*.

**Step 3 .**

Get adjacent pixel set *S*′, which is adjacent to pixel *x*.

**Step 4 .**

Start a loop from here.

**Step 5 .**

Set tag* MeetConstraint* to false.

**Step 6 .**

If *S*′ is not null and tag* MeetConstraint* is false, start a new loop from here.

**Step 7 .**

Search and find a pixel *x*′ in *S*′, which is most similar to* S*.

**Step 8 .**

Herein, there are 2 conditions. One is if hue of *x*′ is similar to that of *S*, or if hue of *x*′ is similar to tongue coating and *x*′ is surrounded by tongue substance pixels. The other one is if the intensity of *x*′ is high. If the 2 conditions mentioned above are met, execute the following 5 steps.

**Step 9 .**

Set tag* MeetConstraints* to true.

**Step 10 .**

Add pixel *x*′ to target region *S*.

**Step 11 .**

Remove pixel *x*′ from pixel set *S*′.

**Step 12 .**

Add those pixels adjacent to *x*′ but not in *S* into *S*′.

**Step 13 .**

Exit the loop which starts from Step 6.

**Step 14 .**

If the conditions mentioned in Step 8 are not met, remove pixel *x*′ from pixel set *S*′.

**Step 15 .**

Loop from Step 4 to Step 14 until tag* MeetConstraint* is equal to false.

In the algorithm described above, color and space information are fully utilized. Therefore, it can lead to a better segmentation result, even if there is a thick coating with different colors on the region of tongue body.

## 3. Results and Discussion

### 3.1. Accuracy Comparisons

**Figure 9:**
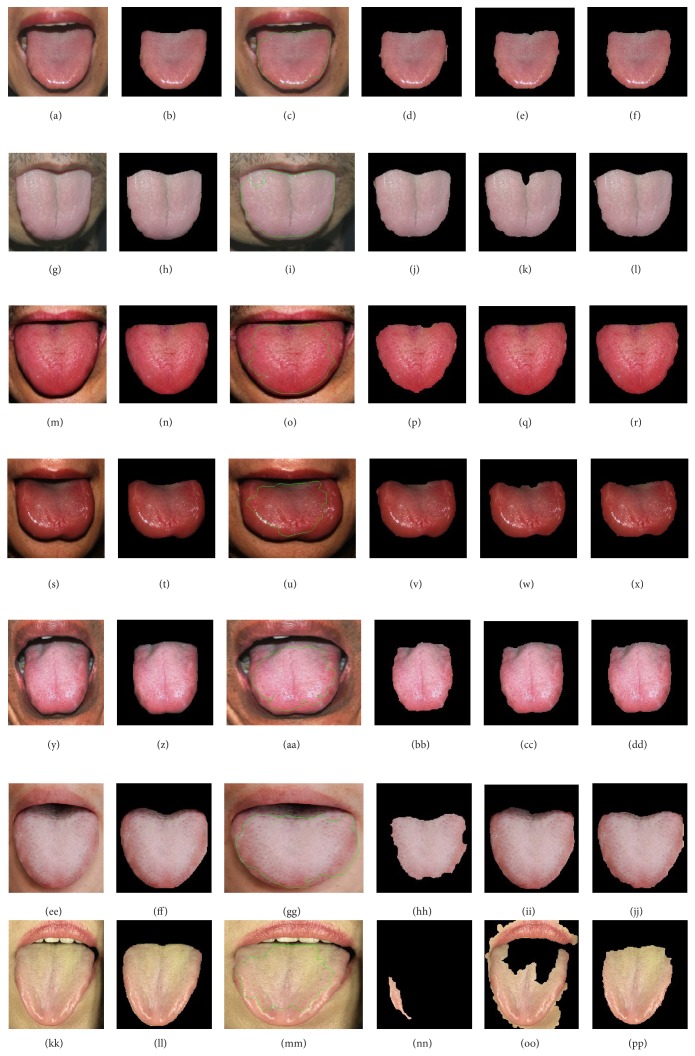
Result comparisons of 4 kinds of tongue image extraction methods. (a) Light red tongue image. (b) Manual segmentation result of light red tongue. (c) Segmentation result of light red tongue image by geodesic active contour. (d) Extraction result of light red tongue by the method mentioned in [21]. (e) Extraction result of light red tongue by the method mentioned in [22]. (f) Extraction result of light red tongue by our method. (g) Light white tongue image. (h) Manual segmentation result of light white tongue. (i) Segmentation result of light white tongue by geodesic active contour. (j) Extraction result of light white tongue by the method mentioned in [21]. (k) Extraction result of light white tongue by the method mentioned in [22]. (l) Extraction result of light white tongue by our method. (m) Red tongue image. (n) Manual segmentation result of red tongue. (o) Segmentation result of red tongue by geodesic active contour. (p) Extraction result of red tongue by the method mentioned in [21]. (q) Extraction result of red tongue by the method mentioned in [22]. (r) Extraction result of red tongue by our method. (s) Deep red tongue image. (t) Manual segmentation result of deep red tongue. (u) Segmentation result of deep red tongue by geodesic active contour. (v) Extraction result of deep red tongue by the method mentioned in [21]. (w) Extraction result of deep red tongue by the method mentioned in [22]. (x) Extraction result of deep red tongue by our method. (y) Purple tongue image. (z) Manual segmentation result of purple tongue. (aa) Segmentation result of purple tongue by geodesic active contour. (bb) Extraction result of purple tongue by the method mentioned in [21]. (cc) Extraction result of purple tongue by the method mentioned in [22]. (dd) Extraction result of purple tongue by our method. (ee) Tongue image with thick white coating. (ff) Manual segmentation result of tongue with thick white coating. (gg) Segmentation result of tongue with thick white coating by geodesic active contour. (hh) Extraction result of tongue with thick white coating by the method mentioned in [21]. (ii) Extraction result of tongue with thick white coating by the method mentioned in [22]. (jj) Extraction result of tongue with thick white coating by our method. (kk) Tongue image with thick yellow coating. (ll) Manual segmentation result of tongue with thick yellow coating. (mm) Segmentation result of tongue with thick yellow coating by geodesic active contour. (nn) Extraction result of tongue with thick yellow coating by the method mentioned in [21]. (oo) Extraction result of tongue with thick yellow coating by the method mentioned in [22]. (pp) Extraction result of tongue with thick yellow coating by our method.

In order to describe the accuracy of our method with fusion of color and space information, we implemented 4 typical kinds of color tongue image extraction methods and compared the results of manual segmentations with the results of these 4 methods. These 4 color tongue image extraction methods are geodesic active contour mentioned in [1], the method based on mathematical morphology and HSI color model mentioned in [21], the method based on sequential algorithm and HSI color model mentioned in [22], and our method with fusion of color and space information suggested in a recent China invention patent. The first method which is geodesic active contour method is implemented with Matlab and the other 3 methods are implemented with VC++. Seven typical kinds of tongue images were taken into account, which are light red tongue, light white tongue, red tongue, deep red tongue, purple tongue, tongue with thick white coating, and tongue with thick yellow coating. All the experimental samples are taken by digital cameras manually under natural light circumstances. And to reduce the processing time costs, all the original tongue images are shrunk to a certain size. Because the colors of the former 5 kinds of tongue image are 5 typical tongue colors in clinical tongue diagnoses and the last 2 kinds of tongue images with thick coating are of important and obvious clinical meanings, the comparison experiment is quite convincible. The segmentation results of these tongue image extraction methods are shown in Figure 9.

As we can see from Figure 9, the results of geodesic active contour are not ideal and the contour curves do not fit well with the boundary of tongue body. Because geodesic active contour takes intensity as the main feature, its segmentation results are greatly affected by the texture of the tongue body surface. For the rest of Figure 9, as we can see, the rest 3 kinds of tongue image extraction methods achieve fairly good segmentation results, except for the last two tongue images with thick coating. As we can see from Figure 9(hh) and Figure 9(nn), the segmentation results of the method mentioned in [21] are quite wrong. This is because the hue color of tongue body surface is not homogeneous. As we can see from Figure 9(oo), the segmentation result of the method mentioned in [22] contains not only tongue body region but also mouth lip region, which is absolutely wrong. This is because the hue and intensity of the tongue body region and the mouth lip region are quite similar. As we can see from the last column in Figure 9, which are the segmentation results of our method, gratifying results have been achieved and all the tongue image segmentations achieve quite good effects. This is owing to the design of fusion of color and space information in our method.

To evaluate the effects of the results of 4 kinds of tongue image extraction methods objectively and quantitatively, we introduce 2 measurement values. One is recognition rate and the other is error rate. The recognition rate *η* and error rate *ε* can be denoted as follows:

(4) $$\begin{array}{c} \eta =\frac{\text{TP}}{\text{TP}+\text{FN}},\\ \varepsilon =\frac{\text{FP}}{\text{FP}+\text{TP}}. \end{array}$$

Herein, TP is the number of pixels which are correctly recognized as tongue pixels, FN is the number of pixels which are tongue pixels but incorrectly recognized as background pixels, and FP is the number of pixels which are background pixels but incorrectly recognized as tongue pixels. The recognition rates of 4 kinds of tongue image extraction methods are shown in Table 1 and the error rates of 4 kinds of tongue image extraction methods are shown in Table 2.

As we can see from Tables 1 and 2, the recognition rates of geodesic active contour method for light red tongue, light white tongue, and tongue with thick white coating are above 80%. But for deep red tongue, purple tongue, and tongue with thick yellow coating, the recognition rates are lower than 70%, except the error rate of the first method for light red tongue which is up to 7.17% and the error rates of the first method for the other tongue images are quite low. The recognition rates of the method based on mathematical morphology and HSI for tongue images without coating are quite high, but for the tongue images with thick coating the recognition rates are quite low. When it comes to the tongue with thick yellow coating, the recognition rate of the second method mentioned in [21] is even lower than 6%. And as we can see from Table 2, the error rates of the second kind of method are lower than 2%. The recognition rates of the method based on sequential algorithm and HSI for most types of tongue images are above 90%, except that the recognition rate for the tongue with thick yellow coating is lower than 75%. And the error rates for most types of tongue images are quite low, except that the error rate for the tongue with thick yellow coating is up to 37.05%. The recognition rates of our method for most types of tongue images are higher than 90%, except that the recognition rate of our method for the tongue with thick yellow coating is a little bit low which is 83.89%. But our method is the most accurate one in these 4 kinds of methods for the extraction of tongue image with thick yellow coating. And the error rates of our method for most types of tongues are not more than 1.2% which is quite low. Generally speaking, as we can see from Tables 1 and 2, the effect of the second method which is the method based on mathematical morphology and HSI is better than that of the geodesic active contour method, the effect of the third method which is the method based on sequential algorithm and HSI is better than that of the second method, and the effect of our method is better than the former 3 kinds of methods in most cases.

### 3.2. Efficiency Comparisons

To show the efficiency of these 4 kinds of tongue image extraction methods, we compare the execution time cost of each method. And the efficiency comparisons are shown in Table 3. As we can see from Table 3, the time cost of geodesic active contour is quite long ranging from 340 seconds to 548 seconds, but the time costs of the other 3 methods are quite low. The time costs of the method mentioned in [21, 22] only cost less than 1 second to fulfill the whole segmentation task. And the time cost of our method is about tens of seconds, which is basically acceptable for the applications of tongue image quantitative checks.

## 4. Conclusions

In this paper, we described in detail 3 kinds of contemporary color tongue image extraction methods based on HSI color model. HSI color model is closer to the perception of human vision, and most of all using HSI color model as the color feature can achieve better segmentation results. Due to the important clinical diagnostic meaning of tongue image with coating, in the case of which traditional tongue image extraction method cannot handle this kind of tongue image well, we suggest a kind of tongue image extraction method with fusion of color and space information, which can handle tongue image with coating quite well. In the experiments, we compare these 4 kinds of tongue image extraction methods, which are geodesic active contour, the method based on mathematical morphology and HSI, the method based on sequential algorithm and HSI, and our method with fusion of color and space information, respectively. As the experimental results show, the effect of geodesic active contour is not very ideal. In most cases, the other 3 kinds of tongue image extraction methods achieve fairly good results. When it comes to the tongue with thick tongue coating, only our method achieves ideal results. In efficiency comparisons of these 4 methods, the efficiency of geodesic active contour is quite low, the efficiency of the method mentioned in [21, 22] is quite rapid, and the efficiency of our method is basically acceptable and practical.

## Figures and Tables

**Table 1:** Recognition rates of 4 kinds of tongue image extraction methods.

Method	Light red tongue	Light white tongue	Red tongue	Deep red tongue	Purple tongue	Tongue with thick white coating	Tongue with thick yellow coating
Geodesic active contour	94.53%	89.16%	71.81%	56.98%	66.81%	83.58%	61.23%
Method based on mathematical morphology and HSI	95.34%	95.71%	88.01%	96.48%	82.99%	67.88%	5.61%
Method based on sequential algorithm and HSI	92.28%	93.15%	96.07%	90.23%	92.45%	95.60%	72.18%
Our method with fusion of color and space information	93.20%	95.66%	95.14%	91.43%	92.02%	93.01%	83.89%

**Table 2:** Error rates of 4 kinds of tongue image extraction methods.

Method	Light red tongue	Light white tongue	Red tongue	Deep red tongue	Purple tongue	Tongue with thick white coating	Tongue with thick yellow coating
Geodesic active contour	7.17%	0.00%	0.00%	0.23%	0.00%	2.26%	0.00%
Method based on mathematical morphology and HSI	0.88%	1.08%	0.00%	1.65%	0.00%	0.00%	0.00%
Method based on sequential algorithm and HSI	0.13%	1.93%	1.35%	0.55%	0.30%	2.03%	37.05%
Our method with fusion of color and space information	0.04%	1.17%	0.08%	0.40%	0.00%	0.40%	0.00%

**Table 3:** Efficiency comparisons of 4 kinds of tongue image extraction methods.

Method	Light red tongue	Light white tongue	Red tongue	Deep red tongue	Purple tongue	Tongue with thick white coating	Tongue with thick yellow coating
Geodesic active contour	406.625 seconds	441.688 seconds	445.641 seconds	447.125 seconds	468.000 seconds	340.922 seconds	548.750 seconds

Method based on mathematical morphology and HSI	0.297 seconds	0.313 seconds	0.297 seconds	0.313 seconds	0.297 seconds	0.312 seconds	0.234 seconds

Method based on sequential algorithm and HSI	0.484 seconds	0.156 seconds	0.171 seconds	0.157 seconds	0.172 seconds	0.157 seconds	0.172 seconds

Our method with fusion of color and space information	14.969 seconds	22.062 seconds	25.375 seconds	18.906 seconds	25.921 seconds	56.829 seconds	15.063 seconds
